# Correcting for measurement error in assessing gestational age in a low-resource setting: a regression calibration approach

**DOI:** 10.3389/fmed.2023.1222772

**Published:** 2023-10-12

**Authors:** George O. Agogo, Jennifer R. Verani, Nancy A. Otieno, Bryan O. Nyawanda, Marc-Alain Widdowson, Sandra S. Chaves

**Affiliations:** ^1^Division of Global Health Protection, US Centers for Disease Control and Prevention, Nairobi, Kenya; ^2^Centre for Global Health Research, Kenya Medical Research Institute, Nairobi, Kenya; ^3^Institute of Tropical Medicine, Antwerp, Belgium; ^4^Influenza Program, US Centers for Disease Control and Prevention, Nairobi, Kenya

**Keywords:** attenuation, fundal height, gestational age, last menstrual period, measurement error, preterm, regression calibration

## Abstract

**Introduction:**

Measurement error in gestational age (GA) may bias the association of GA with a health outcome. Ultrasound-based GA is considered the gold standard and is not readily available in low-resource settings. We corrected for measurement error in GA based on fundal height (FH) and date of last menstrual period (LMP) using ultrasound from the sub-cohort and adjusted for the bias in associating GA with neonatal mortality and low birth weight (< 2,500 grams, LBW).

**Methods:**

We used data collected from 01/2015 to 09/2019 from pregnant women enrolled at two public hospitals in Siaya county, Kenya (*N* = 2,750). We used regression calibration to correct for measurement error in FH- and LMP-based GA accounting for maternal and child characteristics. We applied logistic regression to associate GA with neonatal mortality and low birth weight, with and without calibrating FH- and LMP-based GA.

**Results:**

Calibration improved the precision of LMP (correlation coefficient, ρ from 0.48 to 0.57) and FH-based GA ( ρ from 0.82 to 0.83). Calibrating FH/LMP-based GA eliminated the bias in the mean GA estimates. The log odds ratio that quantifies the association of GA with neonatal mortality increased by 29% (from −0.159 to −0.205) by calibrating FH-based GA and by more than twofold (from −0.158 to −0.471) by calibrating LMP-based GA.

**Conclusion:**

Calibrating FH/LMP-based GA improved the accuracy and precision of GA estimates and strengthened the association of GA with neonatal mortality/LBW. When assessing GA, neonatal public health and clinical interventions may benefit from calibration modeling in settings where ultrasound may not be fully available.

## Introduction

Gestational age (GA) at birth is closely linked with neonatal and infant health outcomes. Accurate assessment of GA is important to guide patient care and public health interventions to improve pregnancy outcomes and infant health (1). More than 60% of deaths in the first year of life are either associated with preterm birth (< 37 weeks of gestation) or with low birth weight (< 2,500 grams) (1, 2). In extremely preterm births, causes of death include respiratory disorders and failure. Mortality rates among preterm infants correlate with birth weight and GA, with low birth weight and preterm births associated with high burden of morbidity and poorer survival. Therefore, infants born with the lowest GA and birth weight have the greatest risk of death (2). Fetal ultrasound is widely used in high-income settings as the gold standard, and biometric measurements provide the most accurate prediction of expected date of delivery, especially if performed before 20 weeks of gestation (3, 4). In low-resource settings, access to ultrasound equipment and skilled technicians is limited, particularly in rural and remote areas (5). In addition, pregnant women in low- and middle-income countries (LMIC) often do not seek prenatal care until later in pregnancy, which further limits the use of ultrasound to assess GA (4, 6). Kenya recommends that a pregnant woman should attend a minimum of four comprehensive personalized antenatal visits spread out during the entire pregnancy as follows: first (< 16 weeks), second (16 to <28 weeks), third (28 to <32 weeks), and fourth (≥ 32 weeks) visit (7). In low-resource settings, such as Kenya, GA is commonly assessed using more affordable but error-prone methods such as the last menstrual period (LMP) or fundal height (FH) (8, 9). Dating based on LMP can be unreliable due to imperfect maternal recall and variable timing of fertilization relative to ovulation (1, 8, 10). Similarly, the accuracy of FH may be affected by multiple pregnancies, maternal size, intrauterine growth restriction, and fetal position (3). Inaccurate GA measurements using these error-prone methods can over- or underestimate the burden of preterm births, which can, in turn, impact clinical care, health services planning, and health policy decisions (11). Moreover, the measurement error in LMP and FH will bias associations of GA with health outcomes and reduce statistical power to detect significant associations (10).

Studies have focused mainly on assessing the validity and accuracy of alternative GA assessment methods relative to the ultrasound (12, 13). Currently, there is limited focus on adjusting for the measurement error in GA before associating GA with a health outcome. We demonstrated a statistical method that corrects for the bias in the parameter estimate that quantifies the association of GA with the health outcome. To demonstrate the method, we used data from a cohort study of pregnant women in rural western Kenya, where GA was measured by ultrasound, FH, and LMP. We used the ultrasound data available from a subset of the women as the gold standard to correct for measurement error in FH- and LMP-based GA in the main study and generated measurement error–corrected GA estimates for all study participants. Subsequently, we used the measurement error–corrected GA values to associate GA with birth outcomes. We also compared FH with LMP regarding their level of precision in measuring GA.

## Materials and methods

### Study population

From January 2015 to September 2019, the Kenya Medical Research Institute (KEMRI) and the Centers for Disease Control and Prevention (CDC) in Kenya conducted a cohort study of pregnant women to assess the burden of influenza disease and its impact on birth outcomes in two rural public hospitals in Siaya County, namely, Siaya County Referral Hospital and Bondo sub-County Hospital (14). The study area has a high burden of maternal and infant mortality, HIV, and malaria, as described in Otieno et al. (14). Pregnancy was confirmed by a blood test or ultrasound, and maternal HIV status was assessed at enrolment, as required by the Kenyan Ministry of Health guidelines, and prophylaxis was provided when indicated. The participants were either recruited during home visits or routine antenatal care visits at the study hospitals. The inclusion criteria included consenting pregnant women at ≤30 weeks gestation, aged 15 to 49 years, resident of a village within 10 kilometers of the study health facility, consenting to HIV counselling and testing, not planning to relocate out of the study area, not enrolled in another interventional study, agreeing to all follow-up visits, and willingness to deliver in the labor ward of the study hospitals (15). The exclusion criteria for the study included multiple pregnancies (twins, triplets, or high-order multiples), history of fistula repair or leg/spinal deformity, or those unable to give informed consent. Participants were followed weekly through either phone calls or home visits until delivery. Structured questionnaires were used to collect demographic and clinical data, including obstetric history, prior pregnancies, medical history, physical examination, and baseline laboratory tests.

### Gestational age estimation

Gestational age was assessed at enrolment by ultrasound, FH, and/or LMP. Ultrasound machines were available initially through routine care as part of the Ministry of Health antenatal care follow-up. However, they were not consistently available to the study staff due to competing priority use for routine care in other services, technician unavailability, or malfunctioning of the machine. In 2018, an ultrasound machine (DP-10 Mindray) was purchased for each study site, and clinicians were trained before using the ultrasound to measure GA. Using this machine, ultrasound GA was calculated according to the parameters obtained in the following measurements: GA in obstetrics (OB) items, average ultrasound age (AUA), and composite ultrasound age (CUA). The GA in the OB items was calculated by the related GA formulae, and AUA is the average of GAs that are calculated according to biparietal diameter, head circumference, abdomen circumference, femur length, gestational sac, and crown rump length, among other parameters. The CUA is calculated using formulae based on biparietal diameter, head circumference, abdomen circumference, and femur length (16). Further details on using this ultrasound machine to measure GA can be found in the operator’s manual (16). Despite that, the ultrasound results were available only for a subset of the study participants. In measuring GA using FH, women were instructed to empty their bladder, a non-elastic tape was used with the graduation in centimeters, and the number of centimeters was considered to correspond to GA in weeks (17). A fundal height was taken as the distance measured from the highest point of the uterus (fundus) to the top of the symphysis pubis. In measuring GA using LMP, the study participants were asked about the date of their last LMP. Last menstrual period was defined as the first day of the last menstruation (4).

### Regression calibration

**Figure 1:**
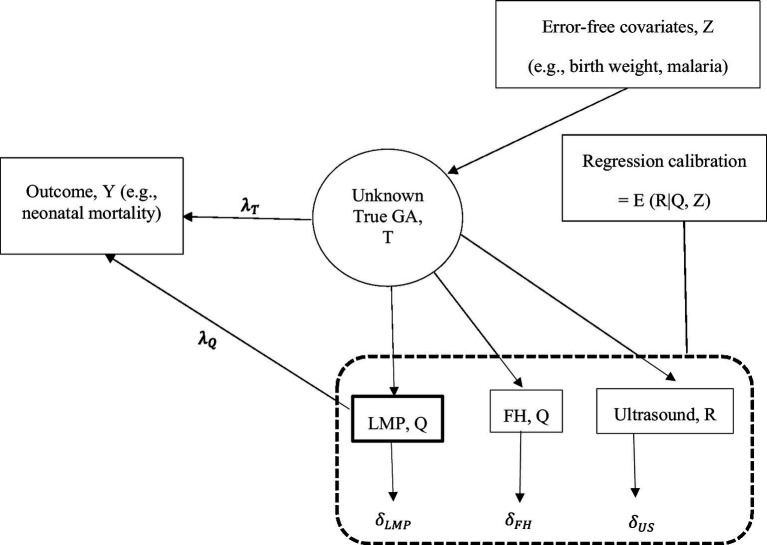
Gestational age (GA) assessment, measurement error, and association of GA with outcome.

We used a regression calibration method to correct for measurement error in FH- and LMP-based GA before relating GA to a health outcome. Using regression calibration, calibrated GA was defined as the conditional expectation of true GA given error-prone FH- or LMP-based GA and predictors of GA assumed to be measured without error (hereafter, error-free predictors) (18, 19). The calibration model was developed from a subset of data derived from the participants with observed ultrasound, LMP, FH, and error-free predictors; then, the resulting GA estimates from the calibration model were used to obtain calibrated GA for all the participants with and without ultrasound measurements. The calibrated GA estimates were then used to associate GA with health outcomes instead of the error-prone measurements from FH/LMP. Using this method, we corrected for measurement error in FH and LMP separately and then jointly. In Figure 1, we denote measurement error in LMP and FH by $\delta_{lmp}$ and $\delta_{fh}$ , respectively, a true parameter that quantifies the association of GA with a health outcome by $\lambda_{T}$ and the biased association parameter by $\lambda_{Q}$ . The regression calibration method adjusts for the bias in estimating the association parameter $\lambda_{T}$ . Using regression calibration, the estimate of the unknown true GA (denoted by T) is a function of GA based on LMP and/or FH (denoted by Q) and error-free predictors (denoted by Z) (18, 20–22). We denoted regression calibration by E (T|Q, Z). Thus, the unknown true association parameter, $\lambda_{T}$ was estimated by using the calibrated GA or the error-prone GA measurements from LMP/FH in the outcome regression analysis (23). Ultrasound-based GA measurement in the calibration model was assumed to be unbiased for true GA and to be measured with random error ( $\delta_{\mathrm{US}}$ ) that is uncorrelated with true GA and measurement error in LMP and FH (18, 19, 24). To calibrate GA based on FH using regression calibration, ultrasound measurements were regressed on FH measurements and a set of error-free predictors of GA. Calibrated GA based on FH was obtained as the conditional mean GA estimates from the calibration model for those with and without ultrasound, provided all other variables were measured and included in the calibration model. To calibrate GA based on LMP and FH jointly, ultrasound-based GA was regressed on LMP- and FH-based GA and a set of error-free predictors.

To adjust for the bias in estimating the association parameter $\lambda_{T}$ , we used the calibrated GA to associate GA with neonatal mortality (deaths within the first 28 days of life, encoded as dead/alive) and low birth weight (< 2,500 grams, yes/no). We modeled the two binary outcomes separately using logistic regression and quantified the association with the logarithm of the odds ratio (hereafter, log OR). To account for the uncertainty in estimating the association parameter due to the calibration process, the standard errors for the calibrated log OR estimates were obtained using the bootstrap method (25–27). With the bootstrap method, the standard error was calculated as the standard deviation of the log OR estimates from the bootstrapped samples. To assess the effect of calibrating GA, we further estimated uncalibrated log OR using GA based on FH and LMP in the logistic regression to associate GA with each outcome.

### Statistical analysis

Gestational age from LMP, FH, and ultrasound was summarized using descriptive statistics. We fitted a linear regression calibration model using ultrasound-based GA measurements as the response under the normality assumption. To eliminate implausible values in the response, we excluded ultrasound measurements ≤0.5th (i.e., 28 weeks gestation) or ≥ 99.5th (i.e., 44 weeks gestation) percentile of the distribution of ultrasound values; these implausible ultrasound values were considered extreme. We assumed the following variables as error-free predictors and included them in the regression calibration model: baby’s birth weight and sex, and for mothers, their age, HIV status, mid-upper arm circumference, malaria and syphilis diagnoses, first pregnancy, education level, trimester of current pregnancy, and hemoglobin levels at enrolment and delivery. The selection of these predictors was guided by their associations with GA at birth from previous studies (28–30). We, however, excluded the baby’s birth weight from the list of error-free predictors when investigating the association of GA with low birth weight. To explore calibrated GA for those with and without ultrasound, we compared their corresponding distributions based on kernel/normal densities and histograms. To quantify the validity of calibrated GA relative to ultrasound-based GA, we used Pearson correlation coefficient, with a value close to 1 implying high validity and a value close to 0 implying low validity. We further used the Bland–Altman method to quantify the agreement between the calibrated GA estimates and ultrasound (31, 32). Using the Bland–Altman method, the discrepancy in the mean GA measurements was quantified by computing the mean of the difference between the calibrated GA and the GA based on ultrasound. A mean difference of 0 indicates no discrepancy in the mean GA values and can be construed as unbiased. The Bland–Altman method further quantifies the precision by constructing 95% limits of agreement. The 95% limits of agreement were calculated as the mean difference ± 2 standard deviations of the mean difference (31, 32). A narrow limit of agreement corresponds to more precise measurements (33). To present the discrepancy in the mean GA measurements graphically, we plotted the difference in GA from the two methods against their mean.

Subsequently, we estimated the association parameter using calibrated GA and hypothesized that the calibration process strengthens the association. To obtain calibrated log OR estimates, we used calibrated GA in the fitted logistic regression analyses that associate GA with neonatal mortality and low birth weight and compared the calibrated log OR estimates with uncalibrated log OR estimates. To obtain the standard error of the log OR estimate, we performed simple random sampling to obtain 1,000 bootstrap samples each of size 1,500. In each bootstrap sample, the log OR was estimated and the standard error obtained as the standard deviation of the log OR estimates from 1,000 bootstrap samples. To assess the effect of ultrasound measurements availability on the performance of regression calibration, we restricted the analysis to pregnant women with all the three GA measurements from ultrasound, FH, and LMP. Subsequently, we created random subsets based on percent ultrasound availability (25, 50 and 100%) by generating a random indicator variable, *r*, using a Bernoulli random number generator, where ultrasound values are set to missing if *r* is zero. Statistical analyses were conducted in SAS software version 9.4 (SAS Institute Inc., Cary, NC). The level of significance was defined as α≤0.05 .

## Results

### Study data description

From January 2015 to September 2019, data for 2,750 pregnant women who enrolled in the cohort study were analyzed (Table 1). The median age was 24.5 years (range: 13.3 to 49.7), and 25.8% of the women were in their first pregnancy. At enrollment, 749 (27.2%) participants were estimated to be in their first trimester of pregnancy, 1,988 (72.3%) in their second trimester, and 13 (0.5%) in their third trimester. Slightly more than half (55.9%) of the participants had attained a primary level of education, and 16% were diagnosed with malaria and 19% with HIV. Among newborns, 51.2% were male, the mean (standard deviation, SD) weight was 3209 (506) grams, and the median weight was 3,210 grams. There were 29 (1.1%) deaths during the neonatal period, and 145 (5.3%) newborns were considered to have low birth weight.

**Table 1:** Descriptive characteristics of study participants from a cohort of pregnant mothers enrolled from January 2015 to September 2019 in Siaya, Western Kenya, *N* = 2,750.

Study variables	Summaries
Maternal age (years)	
Mean (SD)	25.4 (5.5)
Median (range)	24.5 (13.3–49.7)
Maternal education – *n* (%)	
None	27 (0.98)
Primary	1,537 (55.90)
Secondary	850 (30.90)
Tertiary	336 (12.20)
First pregnancy – *n* (%)	
Yes	708 (25.8)
No	2042 (74.2)
Trimester at enrollment (estimated)^a^ – *n* (%)	
First	749 (27.2)
Second	1988 (72.3)
Third	13 (0.5)
Malaria – *n* (%)	
Yes	440 (16.0)
No	2,310 (84.0)
Syphilis – *n* (%)	
Yes	24 (0.87)
No	2,726 (99.13)
Maternal HIV status – *n* (%)	
Positive	513 (18.7)
Negative	2,237 (81.3)
Hemoglobin at enrolment (g/dL)	
Mean (SD)	11.3 (1.7)
Median (range)	11.4 (4.3–19.2)
Hemoglobin at delivery (g/dL)	
Mean (SD)	10.2 (2.3)
Median (range)	10.0 (5.1–18.0)
Maternal mid-upper arm circumference (MUAC) in centimeters	
Mean (SD)	27.3 (3.0)
Median (range)	27.0 (8–45)
Baby’s sex– *n* (%)	
Female	1,341 (48.8)
Male	1,409 (51.2)
Baby’s birth weight (grams)	
Mean (SD)	3,209 (506)
Median (range)	3,210 (1100–5,000)

a Trimester of gestation was estimated by study team at enrollment based on ultrasound, date of last menstrual period, and/or fundal height obtained at enrollment (without calibration).

### Gestational age estimates

**Figure 2:**
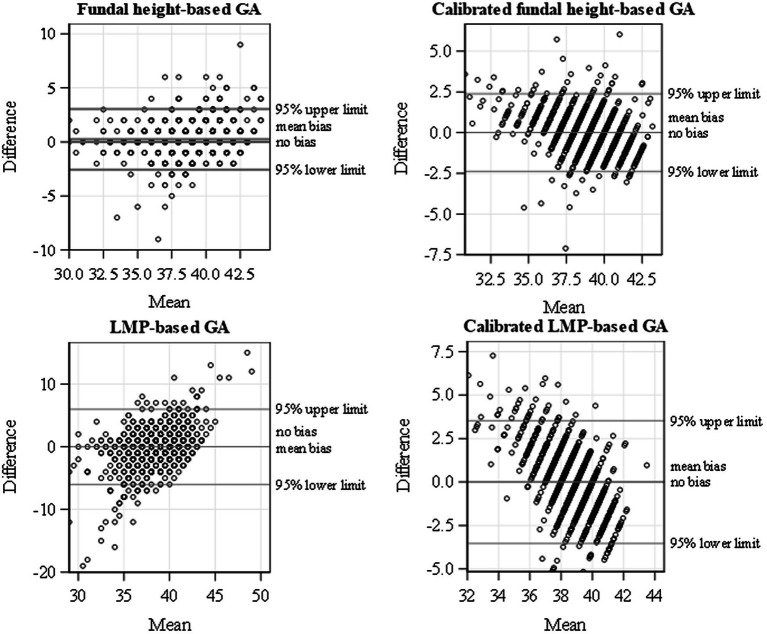
Bland–Altman plot for uncalibrated and calibrated FH and LMP showing the scatterplots of difference in GA and mean with ultrasound and the 95% limits of agreement using data from a cohort of pregnant women conducted from January 2015 to September 2019 in Siaya, Western Kenya.

Ultrasound-based GA data were available for 1,176 (42.8%) pregnant women, FH for 2,521 (91.7%), and LMP for 2,720 (98.9%). The three assessment methods were used together in 1,068 women, constituting 38.8% of all the enrolled women. In fitting the calibration models, we excluded 19 records with potentially extreme ultrasound values. The observed means for GA estimates were 38.7 weeks using the LMP-based method, 38.8 weeks using FH, and 38.6 weeks using the calibrated LMP/FH-based method (Supplementary Table S1). The FH-based mean (SD) GA was 38.8 (2.92), and the calibrated FH-based GA estimate was 38.6 (2.05) weeks gestation. Similarly, the LMP-based mean (SD) GA was 38.7 (3.36) weeks, and the calibrated LMP-based GA was 38.6 (1.27) weeks gestation. The calibrated GA estimates showed less variability (smaller SD) than the uncalibrated GA estimates. The GA obtained by jointly calibrating FH and LMP correlated best with ultrasound (Pearson correlation, ρ=0.84 ), followed by calibrated FH alone ( ρ=0.83 ), and then calibrated LMP alone ( ρ=0.57 ). Calibration improved the correlation between GA based on FH and ultrasound slightly from 0.82 to 0.83 and between LMP and ultrasound from 0.48 to 0.57. Calibrating GA based on FH and/or LMP eliminated the discrepancy in the mean GA estimates relative to the ultrasound-based mean GA (mean difference = 0, Table 2; Figure 2) and improved the precision of the GA estimates (tighter 95% limits of agreement for calibrated GA estimates). Based on the 95% limits of agreement, GA estimated using calibrated FH, on average, may be 2.39 weeks lower/higher than the GA estimated using ultrasound. Similarly, GA estimated using calibrated LMP-based GA on average, may be 3.56 weeks lower/higher than the GA estimated using ultrasound. Thus, the calibrated FH-based GA was more precise than the calibrated LMP-based GA, as shown by the tighter 95% limits of agreement (Table 2). The most precise GA estimates were obtained by calibrating FH- and LMP-based GA jointly.

**Table 2:** Descriptive measures for estimated gestational age (in weeks) at delivery from a cohort of pregnant mothers with paired GA measurements enrolled from January 2015 to September 2019 in Siaya, Western Kenya.

GA assessment methods	*N*	Mean	SD	Median	25th–75th percentile	Minimum maximum	Mean difference^a^ (SD)	*p*-value Ho: bias = 0	95% limits of agreement
Ultrasound	1,176	38.7	2.15	39.0	38.0–40.0	29.0–43.0			
Fundal height (FH)									
Uncalibrated	1,097	39.0	2.45	39.0	38.0–41.0	30.0–47.0	0.25 (1.40)	<0.0001	−2.56–3.05
Calibrated	1,089	38.7	1.79	38.8	37.7–40.0	32.1–44.4	0.00 (1.22)	1.000	−2.39–2.39
Last menstrual period (LMP)									
Uncalibrated	1,154	38.7	3.36	39.0	37.0–40.0	21.0–56.0	−0.01 (3.00)	0.811	−6.00–5.98
Calibrated LMP	1,089	38.7	1.79	38.8	37.7–40.0	32.1–44.4	0.00 (1.22)	1.000	−3.56–3.56
LMP and FH									
Calibrated	1,069	38.7	1.82	38.8	37.6–39.9	32.0–43.9	0.00 (1.19)	1.000	−2.36–2.36

a Mean difference refers to the discrepancy between mean GA estimates from FH/LMP and the mean estimate from the ultrasound; mean estimate from ultrasound is assumed to be unbiased for the true mean GA.

**Figure 3:**
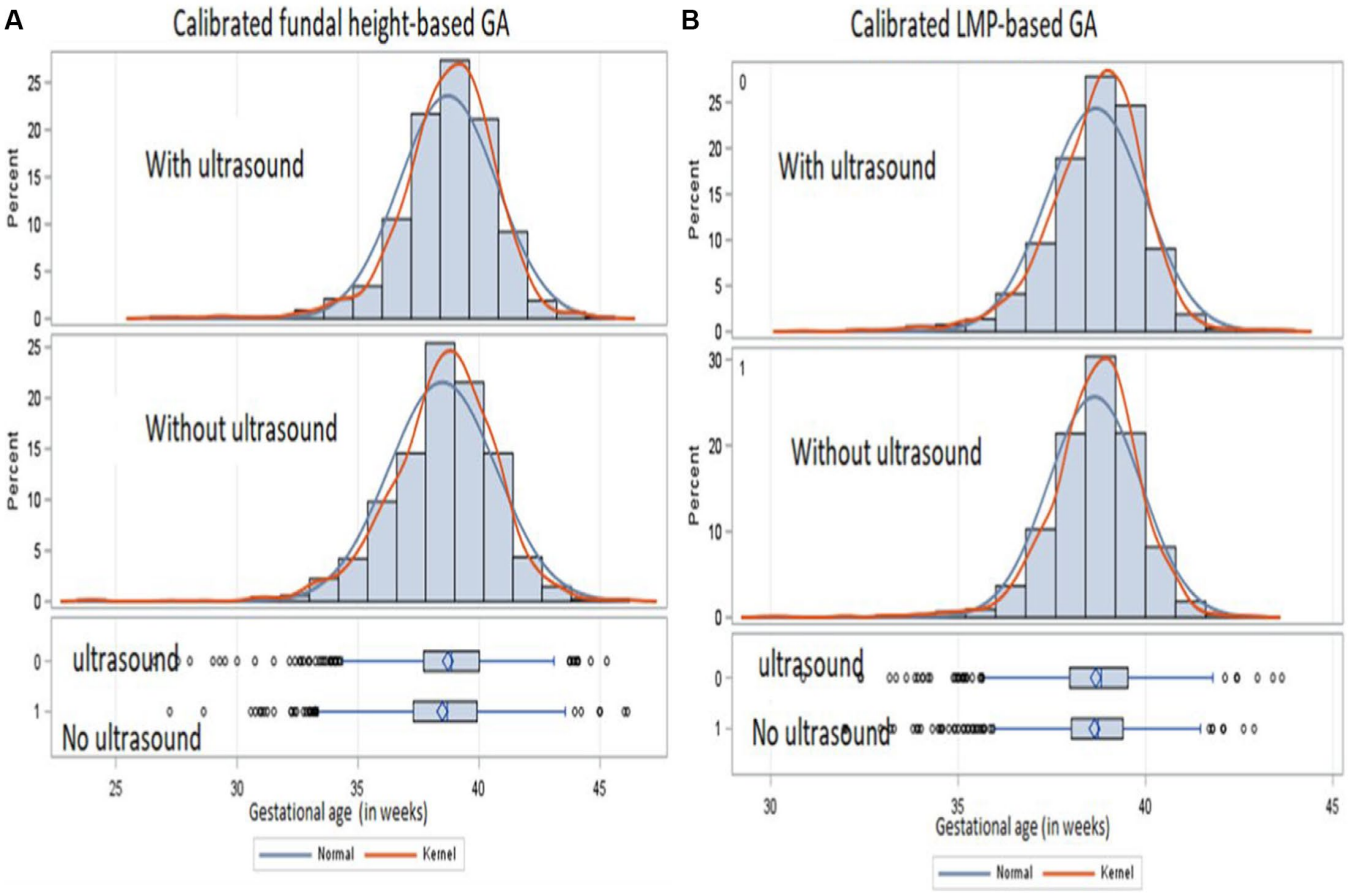
Distribution of calibrated GA from FH **(A)** and calibrated LMP **(B)** for those with ultrasound (upper panel) and those without ultrasound (lower panel) from a cohort of pregnant mothers conducted from January 2015 to September 2019 in Siaya, Western Kenya.

We observed a similar mean distribution of calibrated GA between those with and without ultrasound measurements from the histograms and density plots (Figure 3). However, due to the large sample size, we observed a statistically significant difference in the mean distribution of calibrated FH among those with and without ultrasound (*p* value = 0.001) and of calibrated LMP among those with and without ultrasound (*p* value = 0.009). The percentage of births that would be classified as preterm birth changed from 24.3 to 26.5% by calibrating the FH-based GA and from 19.9 to 11.3% by calibrating the LMP-based GA, suggesting more misclassification when using LMP than FH.

### Association estimates of GA with birth outcomes

In estimating the association of GA with neonatal mortality and low birth weight using continuous GA measurements, the log OR estimates from the calibrated GA were larger, in absolute values, than the log OR estimates from the uncalibrated GA, implying a strengthened association (Table 3). An increase in GA resulted in a significantly lower odds of neonatal deaths regardless of the calibration status of GA measurements. By comparing the calibrated and uncalibrated association estimates for GA with neonatal mortality, the strength of association increased by about 29% (log OR from −0.159 to −0.205) by calibrating GA based on FH, translating into a 3.8% (OR from 0.853 to 0.815) further reduction in the odds ratio of neonatal death. Similarly, we observed an 8.3% (OR from 0.703 to 0.620) further reduction in the odds ratio of neonatal death by calibrating GA based on LMP. We observed a similar result for the low-birth-weight outcome. The smaller log OR estimates (or OR estimate close to one) from the uncalibrated FH- and LMP-based GA implied the attenuation effect of measurement error in GA. The standard error estimates for the log OR that was adjusted for measurement error using regression calibration were larger than the unadjusted estimates due to the uncertainty in calibrating GA. This is further shown by the wider 95% confidence interval (CI) for the calibrated OR estimate. We observed significantly higher odds of neonatal death among preterm births relative to full-term births by calibrating GA based on FH. The odds of a baby having low birth weight were significantly higher among preterm births relative to full-term births regardless of the GA estimation method used. From the sensitivity analysis, the proportion of available ultrasound measurements impacted the calibrated log OR estimates and standard errors, and the impact differed when one measure (FH or LMP) was calibrated compared to when both measures (FH and LMP) were calibrated (Supplementary Table S2). As a general remark, it is advisable to have as many ultrasound measurements as possible to obtain more precise effect estimates.

**Table 3:** Log odd ratio estimates and standard error for the association of gestational age with neonatal mortality and low birth weight using calibrated and uncalibrated fundal height and last menstrual period measurements.

	Association of gestational age with neonatal mortality [*N* = 29 (1.05%)]				Association of gestational age with low birth weight [low birth weight^b^, *N* = 145 (5.27%)]			
GA assessment method	Continuous version		Categorized version (preterm vs. full term)		Continuous version		Categorized version (preterm vs. full term)	
	Log OR (SE)	OR (95% CI)	Log OR (SE)	OR (95% CI)	Log OR (SE)	OR (95% CI)	Log OR (SE)	OR (95% CI)
Fundal height								
Uncalibrated	−0.159 (0.050)	0.853 (0.773;0.897)	0.197 (0.204)	1.218 (0.816;1.816)	−0.353 (0.029)	0.703 (0.664;0.744)	0.669 (0.088)	1.952 (1.643;2.320)
Calibrated	−0.205 (0.090^a^)	0.815 (0.683;0.891)	0.217 (0.321^a^)	1.242 (0.662;2.331)	−0.478 (0.041)	0.620 (0.572;0.672)	0.662 (0.102^a^)	1.939 (1.587;2.368)
Last menstrual period								
Uncalibrated	−0.158 (0.040)	0.854 (0.789;0.889)	0.327 (0.204)	1.387 (0.930;2.069)	−0.273 (0.022)	0.761 (0.729;0.795)	0.860 (0.089)	2.363 (1.985;2.814)
Calibrated	−0.471 (0.097^a^)	0.624 (0.516;0.688)	0.580 (0.216^a^)	1.786 (1.170;2.727)	−0.839 (0.049)	0.432 (0.393;0.476)	0.830 (0.121^a^)	2.293 (1.809;2.907)

a Standard error for the calibrated estimate was calculated using the bootstrap method.

b Low birth weight defined as a birth born with less than 2,500 grams.

## Discussion

Accurate assessment of GA is important for obstetric and pediatric care and guides the timing of interventions at birth in reducing neonatal morbidity and mortality (5). Regarding this, current studies mainly focus on assessing the accuracy of GA assessment tools or on the predicted GA (5, 8). In this study, we demonstrated a statistical method that corrects for measurement error in FH- and LMP-based GA before relating GA to a health outcome. The proposed regression calibration approach uses ultrasound data available in a sub-cohort to correct for measurement error in FH- and LMP-based GA in the main cohort. Calibration improved the correlation between FH/LMP-based GA estimates and ultrasound and eliminated the mean discrepancy in GA estimates relative to ultrasound. Calibrating FH- and LMP-based GA strengthened the association of GA with neonatal mortality and low birth weight, resulting in more valid study conclusions (11, 18, 34). Of note, the calibration parameters may be used to adjust for measurement error in FH and/or LMP in a similar cohort study, provided the parameter estimates from the calibration sub-study are transferable to the cohort to be calibrated (10). However, calibration leads to association estimates with wider CIs due to the uncertainty involved in calibrating GA. Thus, regression calibration does not correct for the loss of statistical power to detect significant associations. Calibration improved the precision of GA estimates, which could affect the estimated prevalence of preterm births as shown in this study, especially when using LMP to measure GA. Low-resource settings may have limited access to ultrasound services, and our analysis provides an alternative way to improve GA estimates when ultrasound results are partially available. The observed higher correlation between FH and ultrasound compared to between LMP and ultrasound is in line with the literature (3, 4), suggesting that FH may be a more precise measure of GA than LMP and could be a better method in routine antenatal care and clinical management (4, 35). Accurate assessment of GA requires proper training of healthcare workers on GA assessment (33). Compared to women with accurate GA to inform the estimated delivery date, women with moderate or severe underestimation of GA may be more likely to miss delivery at the health facility. Notably, measurement error in GA can lead to substantial error in the estimated fetal weight, resulting in misclassification of small-for-gestational-age (SGA) and large-for-gestational-age (LGA) neonates. Small-for-gestational-age neonates are at increased risk of stillbirth and adverse perinatal outcome. Similarly, LGA neonates are at increased risk of perinatal death, birth injury, and adverse neonatal outcomes (36). Measurement error in GA can alter clinical decisions, with either an appropriate-for-gestational-age (AGA) fetus being misclassified as SGA or LGA, an SGA or LGA fetus being misclassified as AGA, or a preterm birth being misclassified as full term. An underestimation of GA can lead to a delay in the induction of pregnancies that have entered the post-term period, increasing the risk of perinatal and neonatal mortality (37). Furthermore, misclassification of GA can impact the decision of whether to administer corticosteroids for lung maturation before the anticipated preterm birth (37). Therefore, there is a need for finding simple but accurate solutions to improve GA estimation in low-resource settings to improve clinical management in providing antenatal care (38). Subsequently, a regression calibration approach can be integrated within the antenatal care system to obtain more accurate individualized GA estimates. This can help clinicians with better classification of fetal weight and preterm births so that neonates can receive appropriate and timely clinical management and medical care (38). Moreover, antenatal care attendance may be limited in LMICs, and some women may not have opportunities for multiple antenatal visits before delivery, as was the case in our cohort study, where only one measurement was available (39).

Although the regression calibration method has been applied in other epidemiologic studies, especially in nutritional epidemiology (18, 20), this study is an innovative application of the method in improving GA estimation. Regression calibration is a relatively simple technique and can be implemented in standard software such as Stata, R, and SAS. In this study, we applied the calibration method to correct for measurement error in error-prone but more affordable GA assessment based on LMP and FH using ultrasound data available only for a subgroup of the study cohort. This approach is important in settings where it may be impossible to measure GA with ultrasound for all pregnant mothers and where multiple measurements throughout gestation may not be feasible due to costs and logistics. For accurate GA estimates from the calibration model, we recommend that ultrasound for estimating GA be conducted at <20 weeks of gestation, at which the biologic variations in fetal size and the effects of growth restriction are still small, and that the calibration model be specified correctly.

Nonetheless, our study has a few limitations. Despite the training of technicians, the reliability of the ultrasound results could have been hampered by inadequate experience, technical expertise of the technicians, and the stage of the pregnancy. In our study, GA was assessed only once, at enrollment, using different methods. Therefore, we were unable to quantify the magnitude of attenuation bias due to measurement error in FH -and LMP-based GA; quantifying attenuation requires multiple measurements per person from each assessment method (5). Additionally, the study participants were from the same ethnicity and geographical region, limiting generalizability to populations with greater demographic diversity or different background rates of health events, e.g., malaria and HIV. Lastly, in applying regression calibration, the additional covariates in the model, such as baby birth weight and trimester, were assumed to be perfectly measured, which may not hold.

## Conclusion

Accurate assessment of GA in low-resource settings poses a challenge to obstetric and pediatric care management. When assessing GA, neonatal public health and clinical interventions may benefit from a regression calibration approach in settings where ultrasound may not be fully available.

## Data availability statement

The datasets presented in this article are available from the corresponding author, GA, ovl6@cdc.gov, after permission from the relevant authority to release the data upon reasonable request.

## Ethics statement

Ethical clearance for the study was obtained from KEMRI Scientific and Ethics Review Unit (KEMRI SSC. 2880) and CDC’s institutional review board (CDC Protocol #6709) in February 2015. Informed consent was obtained from all participants before enrolment in the study. The studies were conducted in accordance with the local legislation and institutional requirements. Written informed consent for participation in this study was provided by the participants’ legal guardians/next of kin.

## Author contributions

GA and SC conceptualized the study objective and the data analytic methods. GA analyzed the data and drafted the first manuscript. BN and NO cleaned the data and wrote the study design section. GA, JV, NO, BN, M-AW, and SC were involved in writing and revising the manuscript. All authors contributed to the article and approved the submitted version.
