# Supplementary material for: Correcting for measurement error in assessing gestational age in a low-resource setting: a regression calibration approach
Source: Front Med (Lausanne). 2023 Oct 12;10:1222772. doi: 10.3389/fmed.2023.1222772 (PMC10613090; doi:10.3389/fmed.2023.1222772)
Supplement: Supplementary file 1 [file Table_1.DOCX]

Supplementary Material

Correcting for measurement error in assessing gestational age in a low-resource setting: a regression calibration approach

**George O. Agogo^1, *^, Jennifer R. Verani^1^, Nancy A. Otieno^2^, Bryan O. Nyawanda^2^, Marc-Alain Widdowson^1,3^, Sandra S. Chaves^4^**

^1^ Division of Global Health Protection, US Centers for Disease Control and Prevention, Nairobi, Kenya, ^2^ Centre for Global Health Research, Kenya Medical Research Institute, Nairobi, Kenya, ^3^ Institute of Tropical Medicine, Antwerp, Belgium, ^4^ Influenza Program, US Centers for Disease Control and Prevention, Nairobi, Kenya.

# *Corresponding Author: George O. Agogo; [ovl6@cdc.gov](mailto:ovl6@cdc.gov)

**Supplementary Table1**. Descriptive measures for estimated gestational age (in weeks) at delivery from a cohort of pregnant mothers enrolled from January 2015 to September 2019, Siaya, Western Kenya

| GA assessment methods | N | mean | SD | median | 25^th^—75^th^  percentile | minimum— maximum |
| --- | --- | --- | --- | --- | --- | --- |
| Ultrasound | 1176 | 38.7 | 2.15 | 39.0 | 38.0—40.0 | 29.0—43.0 |
| Fundal Height (FH) |  |  |  |  |  |  |
| - Uncalibrated | 2521 | 38.8 | 2.92 | 39.0 | 37.0—41.0 | 19.0—49.0 |
| - Calibrated | 2462 | 38.6 | 2.05 | 38.8 | 37.5—40.0 | 23.9—46.1 |
| Last Menstrual Period (LMP) |  |  |  |  |  |  |
| - Uncalibrated | 2720 | 38.7 | 3.36 | 39.0 | 37.0—41.0 | 17.0—56.0 |
| - Calibrated LMP | 2656 | 38.6 | 1.27 | 38.7 | 37.9—39.4 | 29.8—43.9 |
| LMP & FH |  |  |  |  |  |  |
| - Calibrated | 2434 | 38.6 | 2.10 | 38.8 | 37.5—40.0 | 23.2—46.6 |

GA, gestational age; SD, standard deviation.

**Supplementary Table 2.** Calibrated effect estimates and standard errors for the association of GA with neonatal mortality by varying the percent availability of ultrasound used to calibrate fundal height and last menstrual period-based gestational age measurements

| Calibrated method | Percentage of ultrasound availability | | | | | |
| --- | --- | --- | --- | --- | --- | --- |
|  | 25% | | 50% | | 100% | |
|  | logOR | SE | logOR | SE | logOR | SE |
| Calibrated FH | 0.136 | 0.236 | 0.087 | 0.225 | 0.093 | 0.234 |
| Calibrated LMP | -0.077 | 0.218 | -0.083 | 0.193 | -0.135 | 0.218 |
| Calibrated FH & LMP | 0.111 | 0.234 | 0.084 | 0.226 | 0.086 | 0.235 |

logOR, logarithm of odds ratio quantifying the association of gestation age (GA) with neonatal mortality; SE, standard error; FH, fundal height; LMP, last menstrual period.
